# A cluster randomized trial of an organizational linkage intervention for offenders with substance use disorders: study protocol

**DOI:** 10.1186/2194-7899-1-6

**Published:** 2013-12-19

**Authors:** Peter D Friedmann, Lori J Ducharme, Wayne Welsh, Linda Frisman, Kevin Knight, Timothy Kinlock, Shannon Gwin Mitchell, Elizabeth Hall, Terry Urbine, Michael Gordon, Sami Abdel-Salam, Dan O’Connell, Carmen Albizu-Garcia, Hannah Knudsen, Jamieson Duval, Juliane Fenster, Jennifer Pankow, for the CJ-DATS MATICCE Workgroup

**Affiliations:** 593 Eddy Street - Plain St Building Rm. 123, Providence, RI 02903 USA

**Keywords:** Criminal justice, Drug abuse, Treatment services, Organizational change

## Abstract

**Background:**

Substance use disorders are highly prevalent in community correctional populations, yet these settings frequently are ill-equipped to identify and refer offenders to community-based treatment services. In particular, community corrections staff are often opposed to the use of medication in addiction treatment because of inadequate knowledge, resources, and organizational structures to facilitate client linkages to evidence-based services.

**Methods/design:**

Each of the NIDA-funded Research Centers recruited 2 criminal justice agencies to participate in the study. Eligibility rules required study sites that were focused on community corrections (i.e., probation or parole), had few or no formal relationships with treatment providers for referring clients to medication-assisted treatment, and had no state or local policies prohibiting such relationships. Sites under the oversight of the same parent agency were eligible only if they were in geographically distinct catchment areas, and could be assigned to different study arms without cross-contamination at any level. The 18 clusters consisted of community corrections officers and their offender caseloads nested within agencies, each of which was partnered with at least one community-based substance abuse treatment program. Randomization was blocked by Research Center, within which one cluster was randomly assigned to a training-only condition (comparison) and the other to training followed by a strategic organizational linkage process (intervention). Line staff received a scientifically-grounded, systematically-delivered training session that addresses gaps in existing knowledge, perceptions, and information about medication-assisted treatment (MAT) and local availability of MAT services. Key decision-makers subsequently were asked to collaborate in a strategic planning process to enhance formal and informal linkages between criminal justice agencies and local MAT providers. It was hypothesized that the two implementation intervention components together would be more likely than staff training alone to improve the process of referring opioid- and alcohol-dependent adults under community supervision to appropriate addiction pharmacotherapy. Outcomes were measured at the client (referrals), line staff (attitudes), and organizational (linkage) levels.

**Discussion:**

Through closer collaboration among criminal justice agencies and treatment providers, improved linkages to effective substance abuse treatment should yield significant clinical, public health and public safety benefits.

**Trial registration:**

Clinical Trials gov registration number NCT01344122.

## Background

The Criminal Justice Drug Abuse Treatment Studies (CJ-DATS) multisite cooperative is engaged in research on implementing and sustaining evidence-based drug abuse treatment services for persons with substance use disorders involved with the criminal justice system. As such, the CJ-DATS studies seek to determine the effectiveness of one or more *implementation strategies* intended to facilitate the adoption, routine use, and sustainability of evidence-based approaches to the treatment of addiction in offender populations. By facilitating linkages to effective substance abuse treatment through closer collaboration among criminal justice and addiction treatment agencies, significant benefits to public health and public safety are likely to be achieved.

Approximately 4.9 million adults in the U.S. were under parole or probation at year end 2010. (Glaze 2011) It has been estimated that at least 15% of these individuals are opioid-dependent, and alcohol dependence is ubiquitous. (Polcin & Greenfield 2003) While the vast majority of criminal justice referrals to publicly-funded drug abuse treatment programs in the U.S. are through community corrections (Taxman et al. 2007), referrals to addiction pharmacotherapy are rare in most jurisdictions. Substantial evidence supports the effectiveness of medication assisted treatment (MAT) in reducing opioid and alcohol use (Amato et al. 2005; Johnson 2008; Saxon & Miotto 2011; Tompkins & Strain 2011), criminal behavior and arrest (Ball & Ross 1991; Schwartz et al. 2009), and HIV risk behavior and infection (Gowing et al. 2011; Metzger et al. 1993). Moreover, an NIH expert panel (1998), the Office of National Drug Control Policy (ONDCP), and the World Health Organization (WHO) have recommended in the strongest possible terms that opioid agonist treatment be widely available to both criminal justice and non-criminal justice involved populations. MAT for alcohol dependence has been available throughout the world for several decades (Johnson 2008) and has been found to be effective with non-criminal justice populations (Kranzler & Gage 2008; O’Malley et al. 1992; Volpicelli et al. 1992).

While offenders’ lack of insurance coverage for MAT and the prohibitive out-of-pocket cost of these medications are commonly cited as implementation barriers, a planning survey of potential CJ-DATS study sites (Friedmann et al. 2012) identified several additional barriers to the use of MAT with criminal justice populations. One major barrier was the lack of a perceived need for the criminal justice (CJ) system to directly deliver MAT services because addiction pharmacotherapy is available through substance abuse treatment providers within these catchment areas. However, community corrections sites reported few existing linkages with local treatment programs.

A lack of organizational linkages undermines efforts to identify offenders with drug or alcohol dependence for which MAT is indicated, or to refer them to local programs where MAT is available. Facilitating linkages between community corrections agencies and local addiction treatment programs that already offer an array of evidence-based clinical practices would overcome other reported barriers, including concerns about security, liability, and regulatory issues related to storing and prescribing addiction pharmacotherapy within the correctional setting itself. Finally, community corrections staff often lack essential knowledge about the effectiveness of addiction pharmacotherapy, and their philosophical opposition to its use in favor of an abstinence orientation are other frequently cited barriers to the implementation and sustainability of addiction pharmacotherapy in criminal justice settings.

This implementation study protocol addressed a practical problem in real-world settings: FDA-approved medications for the treatment of opioid and alcohol dependence are available in community treatment programs, but are under-utilized. Correctional agencies in those same communities supervise offenders with opioid and alcohol dependence, but lack the capacity to deliver treatment services to them. A lack of communication between corrections and treatment agencies results in a lack of coordination of services that, if delivered, could positively impact both public health and public safety by reducing offenders’ substance abuse and recidivism. The Medication Assisted Treatment Implementation in Community Correctional Environments (MATICCE) protocol addressed interagency collaboration through staff training and coordinated strategic planning. The project did not seek to implement the delivery of addiction pharmacotherapy within community correctional agencies; rather, this study tested the implementation of an organizational linkage intervention (OLI) to increase offenders’ access to already-available evidence-based treatment services in the communities in which they reside. Because the intervention was designed to affect the behavior of community corrections agencies (formalization of referral linkages) and staff (attitudes toward MAT and client referral rates), a cluster randomized design was used.

## Method/design

### Implementation framework

The consolidated framework for implementation research (CFIR) guided the MATICCE study design. (Plsek & Wilson 2001) The CFIR describes 5 domains common across most implementation models: the targeted clinical intervention to be implemented; the internal environment of organizations implementing the practice; the external environment in which those organizations operate; the individuals involved in the implementation process; and the implementation process itself. The MATICCE study protocol addressed these domains as follows:

#### Clinical intervention being implemented

The evidence-based clinical intervention was the appropriate use of medication assisted treatment for clients with opioid and/or alcohol dependence diagnoses. As noted above, the goal of the MATICCE study protocol was to build linkages between community correctional agencies and treatment programs where MAT was already available. Thus the usual implementation concerns about the characteristics of the evidence-based practice (e.g., trialability, observability, relative advantage, etc.) were not the focus of this study because the treatment programs involved in this project had already adopted MAT.

#### External organizational environment

For the criminal justice agencies in this protocol, challenges to linking clients with MAT were nested in the external environment in which these agencies operate. These barriers included a lack of formal relationships with MAT-enabled treatment programs, and limited incentive to negotiate such relationships. Improvement of interagency collaboration and formalization of interorganizational ties were the primary goals of the MATICCE implementation intervention.

#### Internal organizational environment

Relationships between criminal justice agencies and local substance abuse treatment providers may be in part constrained by the characteristics of those agencies – e.g., by an organizational culture that is resistant to collaboration, a low degree of readiness to change current practice, and/or a lack of information systems to support the monitoring of client exchanges between organizations. In this domain, the MATICCE study protocol introduced a strategic planning process to assist probation and parole staff in identifying and overcoming organizational barriers to linking clients with MAT.

#### Individuals involved in implementation

Effectively linking clients with MAT is a task ultimately carried out not by organizations but by individual community correctional staff working in these settings. Staff knowledge and perceptions must be addressed in order to promote acceptance of MAT and its potential role in promoting both public health and public safety. Similarly, information about local MAT resources is needed to permit staff to initiate treatment linkages and to follow up on the progress of individual clients. The MATICCE study protocol included a Knowledge, Perception and Information (KPI) training intervention targeting this implementation domain.

#### Implementation process

Finally, the implementation process itself must be specified and measured. The MATICCE study protocol used a multi-method design (quantitative and qualitative) that allowed for the examination of whether:

- the KPI training + organizational linkage implementation (OLI) strategy increased service coordination and formalization of interorganizational relationships (Aim 1);
- the KPI training + OLI was superior to the KPI training alone in promoting client referral to MAT in the community (Aim 2);
- the KPI training had desired impacts on staff knowledge, perceptions and information about resources relative to baseline (Aim 3); and
- the extent to which the interorganizational linkages were sustained beyond the active intervention period. This domain also includes measurement of the degree to which the implementation intervention was executed as intended (e.g., measures of fidelity/task completion; development of a working alliance between the key participants; and staff satisfaction with the linkage intervention).

### Study settings and participants

Each of 9 CJ-DATS Research Centers partnered with 2 community corrections agencies (probation or parole offices) in distinct geographic catchment areas. Agencies could be under state, federal, or county authority. Agencies within the same parent organization were eligible for inclusion only if they could be randomized in such a way that the participation of one agency in the intervention would not impact the agency assigned to the control group. As part of the MATICCE protocol, the community corrections agencies identified at least one local community-based treatment provider that currently offers medication-assisted treatment, and to/from which the agency would benefit from improving its client flow. If no MAT providers were known to the probation/parole agency, the Research Center staff assisted in identifying and recruiting the participation of a suitable treatment provider organization. Depending on the catchment area and client flow, involvement of multiple treatment provider agencies could be logical and beneficial. Each study site or cluster thus included the community corrections-treatment agency dyad in a given catchment area, the community correctional officers (line staff), and their clients (offenders). MATICCE was tested in 18 study sites located in Arizona, Connecticut, Delaware, Illinois, Kentucky, Maryland, Missouri, New Mexico, Pennsylvania, Puerto Rico, and Rhode Island.

Block randomization occurred by Research Center such that each Research Center contributed one experimental and one control site (cluster) to the study. The requirement for even distribution of sites across Research Centers was a function of the CJ-DATS structure and NIDA grant award requirements. Given the distribution of Research Centers, this effectively meant that there is no more than one experimental site in any single state.

### Protocol phases

The MATICCE study protocol had 4 phases (summarized in Table 1). Although all participating sites were probation and/or parole agencies, jurisdictions varied widely in terms of how these agencies operate, how participants were identified and recruited, and how the data collection for the study was performed. ***Phase 1*** was an initial pre-intervention pilot phase in which the researchers assessed each site and developed, where necessary, any site-specific data collection procedures or methods. For example, sites may have jad somewhat different procedures for storing client records and administrative data; during this initial phase, Research Center staff examined those records and developed a plan for accessing and abstracting each of the protocol’s required data elements. Study investigators reviewed each site’s procedures to ensure consistency of measurement across sites. Phase 1 ended with the collection of baseline survey, interview, and records data from all study sites.

**Table 1:** **MATICCE protocol activities, subjects, and duration**

Project phase	Subjects	Participant burden
***Phase 1: Pilot and Baseline Data Collection (all sites)***		
*Pilot Testing of Data Collection Methods*	Research team	--
*Baseline Data Collection – surveys*	Corrections agency executives and line staff; treatment agency directors and line staff	2 hours
*Baseline Data Collection – interviews*	Key informants (decision makers, line staff)	2 hours
***Phase 2: Training Intervention (all sites)***		
*KPI Training*	Probation and parole officers (line staff)	3 hour session
Presentation and group discussion of science of addiction, available pharmacotherapies, mechanisms of action, evidence base, and appropriateness for criminal justice clients. Delivered by local Addiction Technology Transfer Center (ATTC) staff.		
***Phase 3: Organizational Linkage Intervention Activities (experimental sites only)***		
*Randomization*		
*Formation of Pharmacotherapy Exchange Council (PEC)*	Research team	--
Research team works with CJ and treatment agency leaders to identify and enroll members of the PEC. Includes CJ and treatment co-chairs, a Connections Coordinator, and up to 8 additional individuals from relevant agencies		
*PEC Kickoff Meeting*	PEC members	2 hours
Research team meets with PEC to introduce CJDATS initiative, research aims, MATICCE protocol, timeline, and expectations. Q&A. Higher-ranking agency representatives may also be present.		
*Organizational Linkage Assessment*	PEC members	2 hours, biweekly, 8 weeks
PEC members participate in analyses of agency strengths, weaknesses, opportunities, and threats (SWOT); walkthroughs, and other activities designed to identify areas in which enhanced organizational coordination can better facilitate linkage of drug-involved offenders to treatment programs where MAT is available.		
*Strategic Plan Development*	PEC members	2 hours, biweekly, 8 weeks
PEC uses results of assessment to develop an Organizational Linkage Strategic Plan. Includes identifying high priority process improvement targets, developing action plans, designating responsibilities, establishing timelines. PEC develops a formal written Strategic Plan and submits to agency executives for approval.		
*Strategic Plan Implementation*	PEC members	2 hours, monthly, 7 months
PEC assumes responsibility for implementation of approved Strategic Plan. Assigns roles and tasks to CJ and treatment agency representatives as appropriate; carries out high priority target activities; revises objectives or takes corrective action to ensure objectives are met.		
*Fidelity Monitoring*	Research team	--
Researcher informs/reminds co-chairs of tasks and timelines; observes PEC meetings and records minutes; completes monthly fidelity checklists.		
*Sustainability Planning*	PEC members, Research team	2 hours, 1–2 meetings, 1 month
Researchers convene PEC for formal project wrap-up meeting. PEC assesses relative sustainability of process improvements achieved during implementation phase. Researchers disengage from PEC. PEC may choose to formally disband, meet at less frequent intervals, or maintain current activities.		
***Phase 4: Sustainability (experimental sites only)***		
*Measuring Sustainability*	PEC co-chair, Research team	15 minutes (PEC co-chair)
6 months after disengaging from PEC, research team collects chart abstraction data to identify current rate of client referrals; notes nature and extent of PEC activity (if any) beyond implementation phase. PEC co-chair completes sustainability survey.		

***Phase 2*** involved the delivery of the first component of the implementation intervention – a 3-hour training session designed to address line staff knowledge, perceptions, and information (KPI) about the definition, evidence base, and local availability of MAT for persons with opioid and/or alcohol dependence. The content of the KPI training was developed by the MATICCE workgroup and the Pacific Coast Addiction Technology Transfer Center (ATTC), one of a network of federally-funded training centers that serve as regional information resources on substance abuse treatment. The KPI curriculum drew from a variety of existing resources previously developed by NIDA and by the Substance Abuse and Mental Health Services Administration’s Center for Substance Abuse Treatment (CSAT), grounded in the results of peer-reviewed clinical research. Topics included common myths about MAT and scientific evidence to refute them; a basic overview of brain functioning in relation to drug addiction and MAT; descriptions of the FDA-approved addiction pharmacotherapies and their clinical indications; and advantages that MAT might have for those under criminal justice supervision. The protocol workgroup played an important role in helping shape the presentation to anticipate the needs and interests of a criminal justice audience.

Finally, each Research Center developed an informational pamphlet listing community-based treatment programs in the local catchment area that provdie one or more FDA-approved pharmacotherapies for the treatment of opioid or alcohol dependence. These pamphlets included the name and address of each program, telephone number or other contact information, and a summary of services offered. To reinforce the training content, the pamphlets also included a brief description of each FDA-approved medication and the conditions for which each is indicated.

The KPI training content and its delivery were standardized across all study sites. All probation/parole officers and supervisors in the participating office were invited to attend; research centers had the option to invite staff from other agencies (e.g., law enforcement, treatment providers) as interest warrants. Attendees were consented immediately prior to the training session. Each session began with an introduction by a corrections departmental official stating the facility’s goal of increasing the number of clients who have access to medication assisted treatment. Experienced trainers delivered the KPI curriculum using lecture, question-and-answer, and group discussion methods.

***Phase 3*** began random assignment of criminal justice agencies, blocked by Research Center, to either the experimental (KPI + linkage intervention) or control (KPI-only) group. As each Research Center completed Phase 2, a neutral member of the MATICCE study team assigned clusters to conditions via a coin toss. Research Centers worked with the experimental group sites to identify members for a Pharmacotherapy Exchange Council (PEC), including key decision makers from both the corrections agency and treatment program; line staff from both settings; and other relevant parties depending on local policies and networks. Across study sites, the average PEC roster included 10 members on average [range 6 to 16]. The research team also worked with the study site to identify and recruit a “Connections Coordinator;” that is, a designated person with the skills and networks to facilitate implementation of the plans developed by the group, without serving as the group leader. The PECs were co-led by members representing CJ and treatment agencies.

The organizational linkage intervention fundamentally sought to bridge the “trust gap” that exists between the public health orientation of treatment agencies and the public safety orientation of community corrections agencies. The disconnect between advocacy for the individual in treatment settings and individual accountability in correctional settings sets up a duality of mission that often results in a lack of open communication or even antagonism in the day-to-day workings of organizations that on the surface work together. The PEC took these differences as the starting point for organizational change by focusing on communication. The ability to establish, improve and maintain linkages among community corrections agencies and treatment providers fundamentally depends on building relationships between individuals. The PEC worked to formalize these relationships by establishing objectives, procedures, information sharing protocols, target outcomes, and measures of success that were shaped by both organizations. The PEC engaged in a group strategic planning process to maximize the integration of relationships between community corrections and treatment services, with the ultimate goal of facilitating offenders’ movement between the two systems.

Using a step-by-step manual developed by the MATICCE study workgroup, the researchers trained the PEC members in a strategic planning process that acknowledged the adaptive complexity of the system and mobilized the natural organizing ability and creativity of staff and stakeholders (Plsek & Wilson 2001). The PEC utilized this framework to set goals and expectations to be achieved around the linkage of clients to MAT. Over a 12-month period, the PEC worked through four stages: (a) assessment of existing policies and procedures to identify organizational strengths, weaknesses, opportunities, and threats (SWOT analyses) around MAT service delivery; (b) strategic planning around processes to improve MAT access; (c) implementation of the strategic plan elements; and (d) sustainability planning.

To focus their work, the PEC was charged with identifying no more than 4 high-priority objectives to target in the strategic plan implementation. For example, in the discussion of organizational barriers, the PEC might have discovered that corrections agencies were misinformed about clients’ eligibility for services in local treatment programs; in the strategic planning process, they could focus on developing a strategy to ensure that all probation officers are aware of current admission guidelines. In another site, the PEC might have identified barriers that inhibited the treatment agency from sharing information about a client’s progress with the probation/parole agency; their strategic planning process might have developed a basic information sharing plan, procedures for obtaining appropriate consent, and rules governing content, frequency, and security of information sharing between the two agencies.

During this study phase, the research team provided logistical support (e.g., scheduling meetings) to keep the group on task and to encourage adherence to the overall project timeline. The researchers did not actively facilitate the strategic planning activity other than to guide the PEC back to the steps in the written framework as needed. Investigators from each Research Center met with one another via teleconference bi-weekly to review the project’s overall progress, monitor timelines, and troubleshoot problems as they arose to ensure integrity of the study protocol.

***Phase 4*** represented the PEC’s final task, which was to develop a sustainability plan to guide the participants’ ongoing interactions after the strategic planning period ends. Ideally, sites had jointly adapted local policies and procedures to institutionalize the implemented changes. With a sustainability plan in place, the research team convened the PEC for a final wrap-up meeting, and then formally disengaged from the group. The MATICCE research project period included a 6-month sustainment phase following the active intervention period. After the 6 months elapsed, the researchers returned to the intervention sites to measure sustainment of organizational linkages and client referral patterns.

### Data collection

Data collection activities were designed to address each of the study’s specific aims. Table 2 provides a summary of the data collection instruments used, the sources of data, and the timing of the data collection relative to the protocol’s phases.

**Table 2:** **MATICCE measures, data sources, and timing**

Measures and description	Data source	Timing
*Baseline Survey of Organizational Characteristics:*	CJ agency leadership and probation/parole officers; Treatment program leadership and clinical staff	Baseline
Survey measures organizational climate and culture from leadership and line staff in both CJ and treatment agencies. Includes items to be used as predictors or correlates of implementation outcomes.		
*Interorganizational Relationships Survey:*	CJ agency probation/parole officers; Treatment program clinical staff; assessment agency staff where applicable	Baseline, 12 months
Survey in which staff at each agency rate the quality and frequency of interaction with other agencies involved in MATICCE. At minimum, probation/parole staff and treatment staff rate each other’s organizations. If separate assessment agency is involved in the offender referral process, then their staff also rate, and are rated by, the respective probation/parole and treatment agencies.		
*Opinions about MAT Survey:*	CJ agency probation/parole officers	Baseline, 3 months, 12 months
Survey measures knowledge and perceptions about specific addiction pharmacotherapies, receipt of training, and willingness to refer clients to MAT.		
*Survey of Treatment Referrals:*	Aggregated reports (office-level) from probation/parole agency and treatment program	Monthly from month 1 – 18
Monthly survey obtained from staff or from available information systems at probation/parole and treatment agency. Documents number of offenders referred to the treatment agency by the probation/parole office in the preceding 30 days, and the number of criminal justice-referred clients presenting to the treatment program in the same interval. Supplements agency record abstraction data.		
*Review of Agency Records:*	Offender records maintained by probation/parole offices	Baseline, 12 months, 18 months
Agency records are reviewed to estimate the total number of offenders on agency caseloads during specified intervals, proportion of offenders with indicators of alcohol/drug involvement, and proportion with documented referral to substance abuse treatment. Records are reviewed until 100 alcohol/drug-involved offenders are identified at each interval, or until all records are exhausted. At baseline and 18 months, records are reviewed for the preceding 6 month interval. At the 12-month timepoint, records are reviewed for the preceding 3-month interval. Constructed measures include change over time in the proportion of records with documentation of alcohol/drug involvement and documented referral to treatment.		
*Fidelity Checklist:	Researcher	Monthly from month 1 – 12
The primary research team member assigned to the PEC completes this 30-item checklist each month, indicating whether each of a series of milestones in the Organizational Linkage Intervention has been Not Yet Initiated; Initiated But Not Completed; or Completed.		
*Working Alliance Measure:	PEC members and Connections Coordinator	Monthly from month 1 – 12
This instrument measures the quality of the working relationship between the PEC and the Connections Coordinator. Each rates the other using 16 Likert-type items.		
*Satisfaction with Organizational Linkage Intervention:	PEC members	6 months, 12 months
This satisfaction survey is a 17-item instrument using 5-point Likert items to measure participant perceptions of the organizational benefits and costs associated with participating in the MATICCE intervention.		
*Sustainability Survey:	PEC co-chair	18 months
Measures perceived benefits of the MATICCE intervention, staff engagement in the process, leadership buy-in and organizational structures in place to support continued sustainment of protocol outcomes and processes; collected at 6 months post-intervention.		
*Key Informant Interviews:	4 probation/parole staff + 4 PEC members	Baseline, 12 months
Semi-structured interviews gather information on staff perceptions of interorganizational coordination, its impact on the acceptability of MAT, and the processes involved.		

*Collected from experimental group sites only.

*Baseline Survey of Organization Characteristics (BSOC)* collected from community corrections and treatment managers and line staff measure several important dimensions of organizational structure and climate that may influence implementation outcomes. The BSOC was derived from validated subscales of the Organizational Readiness for Change survey (Lehman et al. 2002) that measure selected domains including as program need for improvement, pressure for change, leadership, staff resources, and training needs; staff perceptions of their opportunities for growth, self-efficacy, influence, and adaptability; and organizational climate scales measuring clarity of mission and goals, staff cohesiveness, openness of communication, and openness to change.

#### Interorganizational relationships (IOR)

Change in the nature and extent of interorganizational coordination was an important implementation outcome. The IOR measure, derived from the work of Van de Ven and Ferry (Van de Ven & Ferry 1980) examined a focal organization’s dyadic relationships with other agencies at baseline and at the end of the organizational linkage intervention period via surveys from agency staff. Probation and parole agents were asked to rate several dimensions of their interactions with the designated treatment agency, while treatment program staff provided companion data. An indicator of MATICCE success (Aim 1) was an increase in the number, type, frequency, and/or formality of these relationships relative to baseline.

#### Opinions and Attitudes about Medication Assisted Treatment (OAMAT)

Instrument assessed attitudes and knowledge regarding methadone, buprenorphine, naltrexone, disulfiram and acamprosate (Springer & Bruce 2008; Gjersing et al. 2007), perceived effectiveness, acceptability, and training received about the medications (Knudsen et al. 2005); and whether each medication should be “used more” and intent to refer (Fitzgerald & McCarty 2009; Varra et al. 2008;) using Likert-type scales.

#### Monthly survey of treatment referrals (MSOTR)

Change in the flow of clients from community corrections settings to local treatment programs was measured by counts of client referrals in two ways (Morrissey et al. 2002). Monthly throughout the intervention period, self-report data were collected from the community corrections agency about the number of offenders identified as having alcohol or opiate use issues, and the number referred to treatment for potential prescription of MAT, as a proportion of the total active caseload. Companion data were collected from the focal treatment program about the number of incoming clients referred from criminal justice sources, the number of those clients with alcohol or opioid problems, and the number who were appropriate candidates for MAT. *Reviews of Agency Records* from community corrections agencies’ client records at three intervals (baseline, 12 months, and 18 months) further assessed changes in referral to MAT. The record abstraction activity reviewed client records for the preceding 6 months and documented the total number of (a) active probationers/parolees in the facility, (b) records with indications of alcohol or opiate involvement, (c) records with indications that the client had been referred for assessment or treatment, and (d) records with indications that the client had received assessment or treatment. While both the self-report and the chart records were imperfect sources for verifying the absolute numbers of client referrals and receipt of treatment, significant changes over time from either or both sources in the intervention group relative to the control group sites would indicate that the intervention had the intended downstream effects on client flow (Aim 2).

#### Implementation fidelity

To the implementation intervention across study sites and over time was captured by three measures. A *Fidelity Checklist* provided time-to-completion data for each of the tasks in the organizational linkage intervention. At monthly intervals, a research team member completed the checklist by indicating whether a series of tasks (e.g., selection of the connections coordinator; training in the application of minimum specifications for the strategic plan) had not started, were partially completed, or were fully completed. These measures assessed adherence to the intervention protocol across sites, measured the time required to complete each step, and related adherence and time-to-completion with site variation in intervention outcomes. Monthly surveys of the PEC membership measured the development of a *working alliance* between the connections coordinator and the PEC. These surveys provided information about how well the group members worked together and whether this facilitated or impeded completion of the component tasks in the linkage intervention as well as the study’s focal outcomes. Finally, *staff satisfaction with the organizational linkage intervention* was measured by surveying the probation and parole officers in each participating study site at 6 months into the linkage intervention and at the end of the implementation phase. These surveys assessed the positive and negative impacts of the intervention on staff, including items such as whether the linkage intervention increased their workload and/or increased their ability to link clients with needed treatment services.

#### Sustainability

The MATICCE study protocol included a 6-month sustainment period to allow the researchers to assess whether any gains in interorganizational linkages and client referrals were sustained beyond the active intervention period. Sustainment was measured using three sources of data. First, the study sites continued to provide Monthly Survey of Treatment Referrals (MSOTR) data on the number of clients referred from the probation/parole agency to the treatment agency, and the number received by the treatment agency from community corrections. Second, the research team again reviewed agency records for documentation of the number of offenders screened, identified, and referred to treatment. Finally, a key informant at each community corrections agency completed a brief *sustainability survey*, which measured the extent to which the organizational linkage intervention and its components were accepted by agency staff, were integrated into ongoing procedures, and continued to inform agency practice.

#### Key informant interviews

In the community corrections agencies and PEC members allowed for a more in-depth understanding of Aims 1 and 3. These semi-structured interviews produced data that speak not only to the acceptability of MAT within the organizational culture, but also to the participants’ process and experience with the organizational linkage intervention. It is possible that the KPI training and the organizational linkage interventions increased either knowledge of MAT or interorganizational coordination, without actually producing marked changes in client referrals as reflected in agency records. In such instances the qualitative interviews help to elucidate the organizational change process that occurred, as well as help to identify any remaining cultural, structural, or organizational barriers (including stigma about drug abuse treatment) that may have persisted. The qualitative interview guides complemented and supplemented the information obtained from the various surveys, thereby providing critical information necessary for triangulating survey and records data for the participating sites at baseline and follow-up time points. Finally, the exploratory nature of the semi-structured interviews created an opportunity to identify and examine unforeseen barriers and facilitators to enhancing treatment services linkages at the participating sites.

### Analysis plan

Randomization occurred at the site level, with the Research Center as a blocking factor. Thus, MATICCE was a multisite cluster randomized trial (Spybrook & Raudenbush 2009). Hierarchical linear modeling (Raudenbush & Bryk 2002) is the preferred analytic method for this design because it accounts for the lack of statistical independence of data (e.g., POs clustered within site within condition) and because it is more flexible than standard statistical regression based on the General Linear Model. Furthermore, HLM can accommodate unbalanced data, thus eliminating the requirement to have the same number of individuals within each cluster. In multilevel modeling, a random intercept or random slope can account for additional outcome variation attributable to individuals and clusters. Finally, a multilevel modeling approach allows for incorporation of covariates for either individuals or cluster levels, which provides the researcher with additional information regarding the nature of the cluster variation.

#### Between-group comparisons

Analyses employed a three-level HLM design for testing the impact of linkage intervention on service coordination, the impact of KPI on staff knowledge and perceptions, and the impact of KPI + OLI on MAT referrals. Sites comprised level 2 clusters that were randomly assigned to either the KPI+OLI or KPI-only group (blocking=level 3), and outcomes were measured at the individual PO level (level 1). The calculated Minimum Detectable Effect Size (MDES) ranges for 3-level MSCRTs reflect that, under the best case scenario of smaller Intra-Class Correlations (ICC) and larger variances explained by blocking and covariates, there was at least 80% power to detect a true population effect as small as 0.37 for hypotheses associated with the study’s specific aims.

#### Within-group comparison across time

A 3-level HLM model was applied to determine the sustainability of the intervention effect for the KPI + OLI group. This model focused on the within-group difference across study phases (repeated measures). Instead of treating time as a continuous variable, which is more common in repeated measure multi-level modeling, two dummy variables representing Phase II, the immediate post-KPI period versus Phase III, the end of the OLI period, and the end of Phase III versus the 6-month follow-up, were created and incorporated as level one covariates. Calculation of the MDES for the 3-level repeated measures HLM model was based on the sample size formula from Teersenstra et al. (Teerenstra et al. 2008). The MDES ranged from 0.34 to 0.46, which corresponds to a medium-small to medium effect size.

## Discussion

The MATICCE protocol proposed organizational- and staff-level implementation interventions to increase the number, formality and effectiveness of interorganizational linkages between community corrections agencies and local treatment providers who offer evidence-based pharmacotherapy for substance use disorders. Improving the strength of these interorganizational ties was hypothesized to increase access to medication assisted treatment services for offenders under community supervision, and thereby reduce their drug involvement and related criminal behavior. At the same time, traditional approaches to real-world implementation (staff training) were employed to address the need for accurate and accessible information. Extensive organizational- and staff-level data collection allowed examination for comparability across the study sites and understanding of the target populations to which the findings apply. The protocol addressed issues of interest to implementation science (organizational- and systems-level interventions; sustainability); health services research (structural influences on treatment access, referral, and service utilization patterns; increased access to MAT); and practical concerns of criminal justice partner agencies (facilitating interagency referrals; reducing relapse and re-arrest rates).

Success in this intervention would address the need to expand access to effective drug treatment services for offenders under community supervision, and may provide a roadmap for facilitating coordination among agencies whose core missions (public health and public safety) are often viewed as incompatible and even adversarial. Likewise, if the combined KPI+OLI intervention shows intended effects relative to the training-only condition, it may provide important evidence for community-based agencies about the limits of staff training as the default approach to implementation of evidence-based practices and system change. Finally, it is hoped that the findings of this study will have practical utility as community corrections agencies seek more efficient and effective ways to manage growing client caseloads in the face of increasingly constrained resources.

## Authors’ information

MATICCE Workgroup Authors include (in alphabetical order): Sami Abdel-Salam (University of Delaware), Carmen Albizu-Garcia (University of Puerto Rico), Lori J. Ducharme (National Institute on Drug Abuse), Jamieson Duval (University of Kentucky), Juliane Fenster (University of Connecticut), Peter D. Friedmann (Rhode Island Hospital and Brown University), Linda Frisman (University of Connecticut), Michael Gordon (Friends Research Institute), Elizabeth Hall (UCLA), Timothy Kinlock (Friends Research Institute), Kevin Knight (Texas Christian University), Shannon Gwin Mitchell (Friends Research Institute), Jennifer Pankow (Texas Christian University), Dan O’Connell (University of Delaware), Lynda A. Stein (University of Rhode Island), Terry Urbine (Arizona State University), and Wayne Welsh (Temple University).
